# Widespread Occurrence of Expressed Fungal Secretory Peroxidases in Forest Soils

**DOI:** 10.1371/journal.pone.0095557

**Published:** 2014-04-24

**Authors:** Harald Kellner, Patricia Luis, Marek J. Pecyna, Florian Barbi, Danuta Kapturska, Dirk Krüger, Donald R. Zak, Roland Marmeisse, Micheline Vandenbol, Martin Hofrichter

**Affiliations:** 1 Department of Bio- and Environmental Sciences, International Institute Zittau, Technische Universität Dresden, Zittau, Germany; 2 Ecologie Microbienne, UMR CNRS 5557, USC INRA 1364, Université de Lyon, Université Lyon 1, Villeurbanne, France; 3 Department of Soil Ecology, UFZ - Helmholtz Center for Environmental Research, Halle/Saale, Germany; 4 School of Natural Resources and Environment, University of Michigan, Ann Arbor, Michigan, United States of America; 5 Unité de Microbiologie et Génomique, Gembloux Agro-Bio Tech, Université de Liège, Gembloux, Belgium; Universidade Nova de Lisboa, Portugal

## Abstract

Fungal secretory peroxidases mediate fundamental ecological functions in the conversion and degradation of plant biomass. Many of these enzymes have strong oxidizing activities towards aromatic compounds and are involved in the degradation of plant cell wall (lignin) and humus. They comprise three major groups: class II peroxidases (including lignin peroxidase, manganese peroxidase, versatile peroxidase and generic peroxidase), dye-decolorizing peroxidases, and heme-thiolate peroxidases (e.g. unspecific/aromatic peroxygenase, chloroperoxidase). Here, we have repeatedly observed a widespread expression of all major peroxidase groups in leaf and needle litter across a range of forest ecosystems (e.g. *Fagus*, *Picea*, *Acer*, *Quercus*, and *Populus* spp.), which are widespread in Europe and North America. Manganese peroxidases and unspecific peroxygenases were found expressed in all nine investigated forest sites, and dye-decolorizing peroxidases were observed in five of the nine sites, thereby indicating biological significance of these enzymes for fungal physiology and ecosystem processes. Transcripts of selected secretory peroxidase genes were also analyzed in pure cultures of several litter-decomposing species and other fungi. Using this information, we were able to match, in environmental litter samples, two manganese peroxidase sequences to *Mycena galopus* and *Mycena epipterygia* and one unspecific peroxygenase transcript to *Mycena galopus*, suggesting an important role of this litter- and coarse woody debris-dwelling genus in the disintegration and transformation of litter aromatics and organic matter formation.

## Introduction

Wood and plant litter are the ubiquitous results of terrestrial plant life. Lignocellulosic biomass is dominant in forest ecosystems and is composed of polysaccharides (cellulose and hemicelluloses) as well as of the aromatic heteropolymer lignin derived from woody plants [1]. Entering soils as wood or leaf litter, these biopolymers are steadily degraded by microorganisms, especially fungi [2]. To gain access to energy-rich polysaccharides in lignocellulosic substrates, fungi must overcome the protecting lignin barrier composed of phenyl propanoid units. Fungi, in particular saprotrophic members of the phylum Basidiomycota, have evolved extracellular oxidative enzymes to oxidize lignin and convert its aromatic constituents [3]. Especially the heme-containing peroxidases of the class II peroxidase group are considered truly lignin-modifying enzymes, which are able to oxidize high-redox potential aromatic substrates [4]. Members of this family of fungal secretory peroxidases, namely lignin peroxidase (LiP, EC 1.11.1.14), manganese peroxidase (MnP, EC 1.11.1.13) and versatile peroxidase (VP, EC 1.11.1.16), are the primary agents of enzymatic lignin decomposition [3]–[6]. In recent studies, two other secreted fungal heme peroxidase (super)families have additionally come into focus, namely the dye-decolorizing peroxidase (DyP, EC 1.11.1.19) and the unspecific or aromatic peroxygenase (UPO/APO, EC 1.11.2.1, a member of the heme-thiolate peroxidase (super)family, which also includes chloroperoxidase, CPO; [6]). DyPs are able to catalyze the efficient oxidation of recalcitrant dyes and aromatics, but do not oxidize Mn like MnPs or VPs [7]. UPOs catalyze a broad spectrum of peroxide-depending oxyfunctionalization reactions, e.g. the epoxidation/hydroxylation of aromatic rings and alkyl chains, ether cleavages, as well as alcohol, aldehyde and phenol oxidations [6], [8]. Although the aforementioned enzymes have been identified and characterized, we do not understand the extent to which they are deployed by saprotrophic fungi residing in forest soils, or their explicit biological function in the detritus decay process.

**Figure 1:**
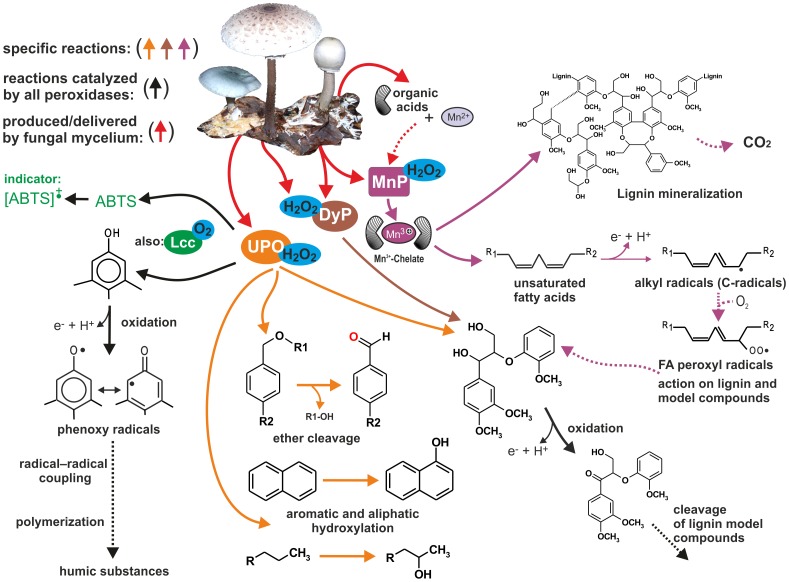
Reactions catalyzed by peroxidases of litter-decomposing fungi found in this study (based on and modified according [48]–[51]). Colored arrows indicate specific reactions of particular peroxidases. Black arrows in the left panel relate to reactions catalyzed by all peroxidases as well as laccase (Lcc). Members of the class II peroxidases not found expressed in litter samples were excluded (LiP, VP).

In general, all three major groups of secreted peroxidases, as well as fungal laccase (EC 1.10.3.2, member of the multicopper oxidase family, [9]), display activities on lignin model compounds and catalyze, in dependence on the reaction conditions, also unspecific polymerization reactions (Fig. 1). Thus, apart from decomposition of wood or litter, peroxidases may be also involved in humification processes in soils [10]–[12] and are therefore of general significance for understanding terrestrial carbon cycling and sequestration. To detect the presence of secreted enzymes such as peroxidases in forest soils, two principal methods are available: i) the measurement of their enzymatic activities in soil extracts (e.g. [13]) or ii) their detection and analysis as mRNA transcripts in the environment [14], [15]. The latter molecular approaches provide useful information about the functional ecological traits, for example, distinguishing fungal activities of saprotrophic litter-decomposing *vs.* wood-rot *vs.* mycorrhizal species, thus providing evidence on the potential impact of particular species and ecological groups regarding lignin decomposition and humus formation. Bödeker and coworkers [16] were able to amplify several class II peroxidase gene fragments, putative MnPs, from genomic DNA of ectomycorrhizal species and inferred a certain potential of these fungi for the decomposition of lignin and related aromatics. This finding has contributed to the ongoing debate about the actual “saprotrophic capabilities” of ectomycorrhizal fungi and their possible impact on ecosystem processes like lignocellulose degradation [17], [18].

Currently only one preliminary study is available following the transcript-level expression of fungal peroxidases in forest litter [14]. In this study, the phylogenetic relationships within the different peroxidase (super)families were not analyzed in depth, especially not within the class II peroxidases. Here, our study aims at the detection and analysis of the above described three major fungal (super)families of secretory peroxidases (including class II peroxidases) in different forest ecosystems that are widespread across Europe and North America. In this context, we address following questions: i) to what extent are the three groups of fungal secretory peroxidases transcriptionally expressed in litter of diverse forest types, and ii) can we identify key fungal species expressing relevant types of peroxidases *in situ*?

## Materials and Methods

Field work permits for the Hainich site in Germany were issued by the responsible environmental offices of the Free State of Thuringia (according to § 72 BbgNatSchG). Samples from the Breuil and Puéchabon sites in France were collected in the framework of collaborative projects including either Dr. Jacques Ranger (INRA Nancy) or Dr. Richard Joffre (CEFE, CNRS, Montpellier), in charge of the Breuil and Puéchabon sites respectively. For remaining sites (Vielsalm, Belgium; Steigerwald, Germany; Oceana and Rhinelander, USA) no permissions were needed, and the field studies did not involve endangered or protected species. The location of sampling sites is given in Table 1.

**Table 1:** Research sites and sampling parameters.

Site	Forest type & treatment	Location, coordinates, altitude	Sampling date	Soil type	Litter C∶N	Litter pH	Soil peroxidase activity in mU g^−1^ DM (substrate)^7^	References (Site, enzymatic data)
1	Sugar Maple (*Acer saccharum*), ambient and control^1^	Oceana Co., Michigan, USA; (43° 40′N, 86° 09′W, 260 m)	Nov 2007	sandy, a mesic Entic Haplorthod	O_e_: 138, A: 55	4.6	up to 25 (L-DOPA)	[46]
2	Aspen (*Populus tremuloides*) and Aspen-Birch (*Populus tremuloides*-*Betula papyrifera*), control plots^2^	Rhinelander, Wisconsin, USA; (45°40.5′N, 89°37.5′W, 450 m)	Oct 2007	sandy, Alfic Haplorthods	∼13 (soil)	5.5 (soil)	up to 15 (L-DOPA)	[52], [53]
3	Beech (*Fagus sylvatica*)^3^	Vielsalm, Belgium; (50°18′N, 5°59.5′E, 450 m)	Oct 2009	Dystric Cambisol	22	n.a.	n.a.	[54]
4	Beech (*Fagus sylvatica*)^4^	Breuil-Chenue, Burgundy, France; (47°18′10″N, 4°4′44″E, 640 m)	July 2007	Alocrisol	19	3.9	0	[55]
5	Beech (*Fagus sylvatica*)	Steigerwald, Ebrach, Germany; (49°52′N, 10°27.5′E, 460 m)	Dec 2008	Dystric Cambisol	20	4.2	n.a.	[56]
6	Beech (*Fagus sylvatica*); natural forest, age-class, plenter forest^5^ (single-tree selection cutting)	Hainich, Mühlhausen, Germany; HEW4-12 (HEW11: 51°06′N, 10°24′E, 400 m)	Mar 2011	Luvisol	33	5.1	90–166 (ABTS), 38–149 (Mn^2+^)	[57]
7	Spruce (*Picea abies*)^5^	Hainich, Mühlhausen, Germany; HEW1-3 (HEW2: 51°12′N, 10°22′E, 375 m)	Mar 2011	Luvisol	29	4.7	162 (ABTS), 610 (Mn^2+^)	[57]
8	Spruce (*Picea abies*)^4^	Breuil-Chenue, Burgundy, France; (47°18′10″N, 4°4′44″E, 640 m)	July 2007	Alocrisol	20	3.9	16 (ABTS), 660 (Mn^2+^)	[55]
9	Oak (*Quercus ilex*); drought and control plots^3^^,^ 6	Puechabon, France (43°44′29″N, 003°35′45″E, 270 m)	Apr & Dec 2010	Shallow, clay over limestone	17	6.3	21–47 (ABTS), 126–374 (Mn^2+^)	[58]

1 Michigan Gradient Study; http://www.webpages.uidaho.edu/nitrogen-gradient/default.htm.

2 FACTSII, Aspen-FACE; http://www.nrs.fs.fed.us/disturbance/climate_change/face/.

3 CarboEurope-IP field site; http://www.carboeurope.org/.

4 SOERE F-ORE-T experimental site; http://www.gip-ecofor.org/f-ore-t/breuil.php.

5 Biodiversity Exploratories; http://www.biodiversity-exploratories.de/.

6 Puéchabon experimental site; http://www2.cnrs.fr/sites/communique/fichier/fluxletterlight_1.pdf.

7 measured using substrate and peroxide, phenol oxidase activity without peroxide was subtracted.

n.a. not available.

### Study sites and litter sampling

Forest floor samples (mixture of O-horizon and leaf litter, excluding Oi horizon) were taken from nine forest ecosystem sites including several subplots in the US and Europe (Table 1). In each plot a minimum of 10 samples were taken, pooled and mixed. About 50 g mixed sample was transported in liquid nitrogen to the laboratory. The 10 randomly selected soil cores covered areas of approximately 100–200 m^2^. The sampling sites represent forests widespread across the investigated areas, dominated, for example, by *Acer saccharum*, *Populus tremuloides* and *Betula papyrifera*, *Fagus sylvatica*, *Picea abies* and *Quercus ilex*. Some of these sampling sites are well established long-term research exploratories in which ecological manipulations have been undertaken and studied, e.g. increased nitrogen deposition, different forest management types, and drought treatments (Table 1 and included references and internet links).

### RNA extraction and cDNA synthesis

A previously published protocol [19] was used to extract total RNA from the different composite forest floor samples obtained from the different research sites. Briefly, the RNA of one gram of forest floor was extracted. One 2 ml screw cap tube contained: 0.25 g sample, 0.5 g glass beads (106 µm, Sigma, Taufkirchen, Germany), 167 µl 3% diatomaceous earth solution (Sigma), 33.3 µl 20% sodium dodecyl sulfate and 583 µl Tris-buffered phenol-based solution (250 g of phenol in 75 ml of 0.5 M Tris–HCl, pH 8, 0.1% (wt/vol) 8-hydroxychinolin, and 0.2% (vol/vol) 2-mercaptoethanol). The sample was disrupted with the FastPrep FP120A instrument (MP Biomedicals, Solon, USA) for 30 s at a speed of 6.5, or alternatively with a Dismembrator (Sartorius, Göttingen, Germany) at 2,000 rpm for 2–3 min. The crude mix was then centrifuged, the nucleic acids precipitated with ethanol and sodium acetate (0.3 M), and the RNA purified using the Nucleobond RNA/DNA 400 kit (Machery-Nagel, Düren, Germany) as recommended by the manufacturer. Before further purification of the RNA with the RNeasy Plant Mini kit (Qiagen, Hilden, Germany), an extra DNAse step (Qiagen) was carried out as recommended by the manufacturer. Approximately 500 ng of purified DNA-free RNA was used as template for reverse transcription, after which the cDNAs were amplified *via* 17–19 cycles of a long-distance PCR (LD-PCR) using the SMART PCR cDNA Synthesis Kit according manufacturer protocol (Clontech, Mountain View, USA), hereby specifically enriching the mRNAs of the sample.

### PCR, cloning, sequencing, and phylogenetic analysis of proteins

Degenerate primers for class II peroxidase, i.e. MnP were developed previously by Maijala and coworkers [20] and later by Bödeker and colleagues [16]. In this study, we used a slightly modified primer combination, peroxiF2 (5′-GGY GGI GGI GCB GAY GGY TC-3′) and peroxiR2 (5′-GG IGT IGA RTC GAA BGG-3′), which gave better amplification results (Barbi et al, *unpublished*). UPO was amplified by using APO_65F (5′-AAY GCI ATG GCN AAY CAY GG-3′) and APO_130R (5′-GC RTC RTG YTC IAT NCC-3′; [14]). Lastly, primers for DyP were newly developed and used; DyP360F (5′-TGY CCI TTY GCI GCN CAY AT-3′; protein motif: CPFAAHI) and DyP485R (5′-RAA RAA RTA YTC ICC NCC-3′; protein motif: GGEYFF).

For PCR amplification, in a 25 µl PCR reaction using DreamTaq Green PCR Master Mix (Fermentas, St. Leon-Rot, Germany), 0.25 µl of forward and reverse primer (100 mM, Biomers, Ulm, Germany) and 0.5 µl cDNA template were added. The following program on a Mastercycler thermocycler (Eppendorf, Hamburg, Germany) was used for amplification: initial denaturation for 5 min at 94°C, 35 cycles of denaturation (45 s at 94°C), annealing (45 s at 50°C), and elongation (1 min 40 s at 72°C), followed by a final elongation step for 10 min at 72°C. PCR products of expected sizes (MnP: ∼400 bp, UPO: ∼210 bp, DyP: ∼400 bp) were gel purified, combined among the independent soil replicates of one field site (Table 2), and cloned into the pCR4-TOPO vector using the TOPO TA Cloning Kit for Sequencing according manufacturer protocol (Invitrogen Life Technology, Karlsruhe, Germany). Ten positive clones for each expressed peroxidase and treatment were sequenced at GATC Biotech AG (Konstanz, Germany). Obtained nucleotide sequences were edited with BioEdit 7 [21], translated to protein sequences, and identified with blastp using GenBank database. Protein sequences were aligned using ClustalW and phylogenetically compared with references obtained from fungal cultures, GenBank and PeroxiBase [22] using MEGA 5 [23]. Following parameter for a neighbor-joining analysis were used: substitution model: Poisson, site rates: uniform, gaps: pairwise deletion. For branch support 1,000 bootstrap replicates were performed; only values above 50% were shown. All 173 newly obtained sequences were submitted to GenBank and are available under accession numbers JQ654238–JQ654411 and FR875240–FR875262.

**Table 2:** Number of field site replicates and number of subsequent achieved PCR products is given for each peroxidase type.

Site	Forest type & treatment	# of field site replicates	# PCR products; # unique class II peroxidase genes of PCR pool	# PCR products; # unique UPO genes of PCR pool	# PCR products; # unique DyP genes of PCR pool
1	Sugar Maple, ambient; USA	3	2; 1$	3; 8$	3; 5
1	Sugar Maple, N amended; USA	3	2; 7$	3; 3$	3; 3
2	Aspen, control plots; USA	3	2; 2	3; 6	3; 7
2	Aspen-Birch, control plots; USA	3	2; 2	3; 2	3; 4
3	Beech; Vielsalm, Belgium	3	2; 4	2; 5	0
4	Beech; Breuil-Chenue, France	1	1; 6	1; 8	1; 4
5	Beech; Ebrach, Germany	3	2; 2	2; 3	0
6	Beech, natural forest HEW10-12; Hainich, Germany	3	3; 6	3; 3	0
6	Beech, age-class forest HEW4-6; Hainich, Germany	3	2; 2	2; 2	1; 3
6	Beech, plenter forest HEW7-9; Hainich, Germany	3	3; 3	2; 3	0
7	Spruce, HEW1-3; Hainich, Germany	3	3; 2	1; 4	0
8	Spruce, Breuil-Chenue, France	1	1; 17	1; 4	0
9	Oak, control plots, April; France	2	2; 7	2; 3	2; 6
9	Oak, control plots, December; France	2	2; 3	2; 7	2; 8
9	Oak, drought plots, April; France	2	2; 6	2; 6	2; 7
9	Oak, drought plots, December; France	2	2; 6	2; 7	2; 7
	**Total unique types, based on DNA/protein sequences**		**76/71**	**72/70**	**54/52**

Number of unique sequence types is shown for the pool of PCR products.

$ partially published in [14].

### Peroxidase reference sequences from pure cultures

To increase the number of reference sequences for phylogenetic analyses of the new environmental sequences, we cultured and transcriptionally analyzed peroxidases from following pure fungal cultures: *Agrocybe aegerita* DSMZ 22459, *Agrocybe pediades* CBS 10139, *Clitocybe nebularis* IHI 460, *Collybia tuberosa* IHI 452, *Cortinarius odorifer* CBS 517.95, *Exidia glandulosa* DSMZ 1012, *Gymnopus* sp. IHI 363, *Lycoperdon perlatum* IHI 456, *Macrolepiota procera* IHI 418, *Marasmius rotula* DSM 25031, *Mutinus caninus* IHI 508, *Mycena epipterygia* IHI 195, *Mycena galopus* IHI 376 and *Suillus variegatus* IHI 503 (for strain origin see Table S1). Strains were cultured in wheat straw (3 g straw in 15 ml H_2_O), tomato juice, liquid soy medium, or synthetic liquid Kirk medium supplemented with 100 mg l^−1^ MnCl_2_ [7], [24], [25]. All strains were grown at 24°C as surface cultures for 20–40 d. Mycelia were harvested by carefully removing the mycelial mat from the culture liquid and the total RNA was isolated using Trizol (Invitrogen). Then, the SMART PCR cDNA Synthesis Kit was used to synthesize the cDNA, and PCRs were performed as described above. Obtained PCR products were cloned and sequenced for each fungal strain. All new reference sequences were submitted to GenBank under accession numbers JQ654412–JQ654445.

To test extracellular MnP activity, all strains were cultured on Kirk agar plates supplemented with 100 mg l^−1^ MnCl_2_. Plates of MnP-positive fungi developed characteristic dark-brown to black rings or spots caused by precipitating manganese(IV) oxide (MnO_2_) that, for its part, resulted from the disproportionation of Mn^3+^ (2 Mn^3+^→Mn^4+^+Mn^2+^), formed through the oxidation of Mn^2+^ by MnP [24].

### Peroxidase activity in litter

Briefly, 10 g of a forest floor sample from selected forest sites and 100 ml distilled water were shaken for 2 h at 8°C, centrifuged and the supernatant used for enzymatic measurements. All enzyme assays were prepared in a total volume of 1 ml including 50 µl of the enzyme-containing extract. Enzyme activities were recorded in 1 ml cuvettes using a Cary 50 Bio UV-Vis spectrophotometer (Varian, Palo Alto, CA). For manganese independent peroxidase (MiP) activity, a 2-step protocol was performed. First, laccase (phenol oxidase) was determined spectrophotometrically with ABTS (2,2′-azino-*bis*(3-ethylbenzothiazoline-6-sulphonic acid), *ε*_420_ = 36,000 M^−1^ cm^−1^; 0.3 mM) in sodium-citrate buffer (50 mM, pH4.5) and the reaction was followed at 420 nm for 2–3 min. Then, second, MiP activity was measured in the same assay mixture after addition of 0.1 mM H_2_O_2_ and by following the reaction at 420 nm for another 2–3 min; the final MiP activity was calculated by subtraction of the laccase activity [26]. Manganese dependent peroxidase activity was determined in sodium-malonate buffer (50 mM, pH 4.5) amended with MnCl_2_ (0.5 mM). The reaction was initiated with 0.1 mM H_2_O_2_ and followed at 270 nm (*ε*_270_ = 11,590 M^−1^ cm^−1^) for the formation of Mn^3+^ malonate complexes; protocol modified according to Wariishi and coworkers [27]. Enzymatic activity is expressed as unit (U), which is defined as the amount of the enzyme that catalyzes the conversion of 1 micro mole of substrate per minute.

## Results

### Detection of peroxidase transcripts in forest soils and in fungal pure cultures

Throughout this study, three primer pairs were used for PCR amplification of peroxidases from 40 cDNAs representing replicates of nine different research sites and included subplots (Tables 1 & 2). Using class II peroxidase primer, 36 of the 40 (90%) cDNAs/samples showed a PCR product of the correct basepair length. UPOs were amplified in 34 (85%) samples and DyPs in 22 (55%) samples (Table 2). In several cases, unspecific PCR products were recorded, especially by using the class II peroxidase primer (see also [16]; i.e. short amplification products). Altogether 202 unique peroxidase transcripts from environmental samples were analyzed; 76 of the class II peroxidases, 72 of the UPOs and 54 of the DyPs (Table 2). All peroxidase (super)families were transcriptionally detected in each forest litter type, except for DyPs that were not found in spruce litter (Table 3).

**Table 3:** Richness of phylogenetically characterized peroxidase (super)families and groups in different forest ecosystems.

	Transcripts found in forest litter:				
Peroxidase family	Beech	Spruce	Oak	Aspen & Aspen-Birch	Maple
*Class II peroxidases*					
MnP (EC 1.11.1.13, hybrid or unclassified, Group A* or A.2)	19	3	20	4	7
MnP long (EC 1.11.1.13, Group B)	5	13	2	0	1
LiP (EC 1.11.1.14, Group A.1)	2#	1#	0	0	0
VP (EC 1.11.1.16, Group A.3 or subgroup in C)	0	0	0	0	0
GP (EC 1.11.1.7, Group C)	1	2	0	0	0
*Dye-decolorizing peroxidases* (EC 1.11.1.19)	7	0	28	13	8
*Unspecific peroxygenases* (EC 1.11.2.1)	25	9	23	8	11

*sequences include Asp-175 (in *P. chrysosporium* MnP1).

# not appearing in classic group A.1 LiP clade (see Fig. S1).

In an independent approach, partial peroxidase transcripts were amplified in all analyzed cultures (Table S1). Class II peroxidase transcripts were amplified and sequenced in nine fungal strains, DyP in seven, and UPO in ten species, respectively. Dark-brown precipitates in agar plates supplemented with MnCl_2_, providing evidence for MnP activity, were visually recorded after 4–6 weeks of growth for four strains (Table S1).

### Phylogenetic analysis

All new fungal peroxidase sequences were subject to phylogenetic analyses (Figs. S1, S2, S3). A phylogenetic analysis of 80 (76 unique when compared on nucleic acid sequence level, 71 on protein level, Table 2) partial class II peroxidase protein sequences from environmental samples together with 360 reference sequences obtained from fungal cultures, GenBank and PeroxiBase, yielded a distinct clustering structure (Fig. S1). Class II peroxidases were clustered following Lundell and coworkers [4] and Floudas and coworkers [3] into the larger groups A–C, which include, in sub-clades, the different enzymes LiP (group A.1), hybrid MnP or short MnP (hMnP, A.2 or “MnP short”), VP (A.3, and sub-clades), long MnP (group B) and generic peroxidase (group C, “GP”, diverse sub-groups), as well as undefined entries (Fig. S1). Group A.1 (LiP) and group B (“MnP long”, “MnP extra-long”) formed distinct clades, whereas previously defined members of group A.2 (hMnP), A.3 (VP) and C (GP and others) appeared in an undefined structure, showing several sub-clusters or representing not consistent enzyme types. Class II peroxidases from forest samples were recorded largely in group A, which relates to diverse MnPs, several in group B (“long” MnPs), and few in group C (GPs) (Fig. S1). No environmental sequence appeared within group A.1 (LiP) or A.3 (VP) or close to those known LiP or VP sequences.

**Figure 2:**
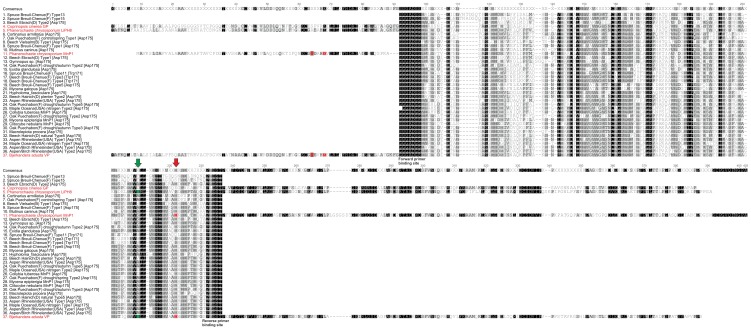
Alignment of class II peroxidase full length reference protein sequences (red) and a selection of partial protein sequences found in this study, representing different clades of the calculated phylogenetic tree. Three acidic amino acid residues marked red in *P. chrysosporium* MnP1 (AAA33744) and *B. adusta* VP (AAO47909) are crucial for Mn^2+^ oxidation, and the tryptophan residue marked green in *P. chrysosporium* LiPH8 (AAA53109) and *B. adusta* VP is responsible for oxidation of phenolic compounds. *Coprinopsis cinerea* GP (CAA50060) does not include these catalytic residues. Red and green arrows mark the catalytically important sites within the amplified sequences.

Additional support for this finding was obtained when the amino acid residues of the partial protein sequences were analyzed in more detail. The majority (96%) of the environmental sequences displayed no putative catalytically active tryptophan residue (Trp-171 in *Phanerochaete chrysosporium* LIPH8, Fig. 2) on alignment position 210, which indicates that these sequences do not encode LiP or VP enzymes. Only three sequences exhibited such a residue, but no aspartic acid residue (Asp-175 in *P. chrysosporium* MnP1; Figs. S1, 2) 12 alignment positions downstream of Trp-171, which would be indicative for a manganese binding site present in MnPs and VPs. Since these three sequences distantly cluster to LiP sequences of group A.1, they were designated as “untypical” LiPs (Fig. S1). Analysis for the putative manganese binding site amplified in our partial protein region, 73 (92%) of the environmental sequences had an acidic aspartic residue (Asp-175 on alignment position 222, Fig. 2), indicating a transcript encoding an enzyme with potential MnP activity.

Despite the large amount of class II peroxidase reference sequences used in the present phylogenetic analysis, only few environmental sequences could be identified (Fig. S1). Sequences from a spruce forest (JQ654274) and a beech forest (JQ654280) could be assigned to a putative MnP sequence of *M. epipterygia* with 100% and 98% amino acid identity, respectively. Furthermore, a second sequence from a beech forest (FR875255) was matched to a reference sequence of *M. galopus* with 99% amino acid identity. A sequence of an oak forest site clustered within a clade consisting of different *Cortinarius* spp. sequences (89% amino acid identity to *C. hinnuleus* ACX51152; Fig. S1). Other environmental sequences could not be related to references with high confidence.

Several protein encoding sequences were found identical in different forest sites or among different subplots. For example, one MnP of group B occurred in a beech forest site in Germany (JQ654246) and an oak forest in France (JQ654264); in two cases, a MnP of group A was found in the oak forest regardless of drought treatment and/or season (i: JQ654253, JQ654260, JQ654266, ii: JQ654258, JQ654272), and finally, another MnP of group A was shared between a single-tree selection cutting and an age-class beech forest site in Germany (JQ654276, JQ654278) (Fig. S1).

Altogether, 76 (72 unique) environmental partial UPO protein sequences were compared to 75 reference sequences from GenBank and data from this study (Fig. S2). The majority of environmental sequences clustered close to basidiomycete references in a larger clade (group I.1, Fig. S2), apart from clades dominated by ascomycete reference sequences (group I.2). However, none of the investigated environmental sequences were found to be closely related to the clade (group II) of *A. aegerita* UPO1 and UPO2 or *C. cinerea* UPO sequences, which contains well-studied UPO (previously assigned APO) enzymes [28]. This finding is supported by large differences in length in the amplified partial protein sequences. Within the amplified region, environmental sequences of group I.1 were generally 66–74 (mean 70) amino acids long, whereas homologous regions in the full sequence of *A. aegerita* UPO1 and UPO2 as well as *C. cinerea* sequences of group II are 80–90 amino acids long. So far, biochemical characteristics based on single characteristic amino acid residues in the amplified region cannot be applied to support a distinct clustering or potential function.

One environmental UPO sequence from a spruce forest in Germany (JQ654347) could be assigned to a reference sequence from *M. galopus* with 100% amino acid identity (Fig. S2), other sequences were not classifiable with high confidence. Similarly to the above described class II peroxidases, several environmental UPO sequences were shared between different forest sites, for example, one UPO-type sequence occurred in beech forests in Germany, France, and Belgium (JQ654300, JQ654305, JQ654332) (Fig. S2).

Fifty-six environmental partial DyP sequences (54 unique) were compared with 95 reference sequences in a phylogenetic analysis. The environmental sequences clustered in several distinct clades, mainly together with basidiomycete reference sequences (Fig. S3). Compared to the other peroxidases, however, assignment of any environmental sequence was not possible with high confidence. All amplified partial DyP sequences showed considerable differences in length, ranging from 128 to 144 amino acids. Within the amplified region, three important amino acid residues forming the binding pocket for H_2_O_2_ can be recognized [29]. The majority (>98%) of the environmental sequences had two of these important positions, namely arginine Arg-329 (alignment position 54) and leucine Leu-354 (position 90) (nomenclature based on the structure of *Bjerkandera adusta/Thanatopherus cucumeris* DyP 3MM3); a phenylalanine Phe-356 (alignment position 92) was present in 91% of the analyzed environmental sequences. However, no further biochemical characterization based on these amino acids residues is possible at the current state, and none of the environmental sequences was found twice in different sampling sites.

### Enzymatic peroxidase activity

Twelve plots (nine beech, three spruce forests) at four sites in the German Biodiversity Exploratory Hainich, as well as all three plots in beech, spruce and oak forests in France were analyzed for potential peroxidase activity (Table 4). Manganese independent peroxidase activities were observed in all plots, except for beech site 4 (France), and ranged from 0 to 166 mU g^−1^ dry matter (DM; Table 4). MnP activities were measured in the range from 0 to 660 mU g^−1^ DM in forest plots (Table 4).

**Table 4:** Extracellular peroxidase activities in selected litter samples (n = 3).

Sampling site	Mn-independent peroxidase activity (mean ± stdev mU g^−1^ DM)	Mn-dependent peroxidase activity (mean ± stdev mU g^−1^ DM)
Beech, natural forest (Hainich, site 6)	166±53	149±70
Beech, plenter forest (Hainich, site 6)	90±50	38±32
Beech, age class (Hainich, site 6)	145±170	138±180
Spruce (Hainich, site 7)	162±50	610±850
Beech (Breuil-Chenue, site 4)	0	0
Spruce (Breuil-Chenue, site 8)	16±1.6	660±88
Oak, control (Puechabon, site 9)	47±2.8	126±179
Oak, drought plots (Puechabon, site 9)	21±1.6	374±530

## Discussion

Fungal secretory peroxidases are known to be of significance during litter decay, inasmuch as they initiate the decomposition of lignin and transformation of resulting aromatics, which, in turn, foster humus formation (Fig. 1; [3], [4], [8], [30]). This is especially true in forest ecosystems, in which lignified plant cell wall enters soil as leaf, needle, root and woody litter. In our survey, we found a widespread transcript-level expression of members from three major (super)families of fungal secretory peroxidases, namely manganese peroxidase (MnP, member of the class II peroxidases), unspecific (previously: aromatic) peroxygenase (UPO, member of the heme-thiolate peroxidase superfamily) and dye-decolorizing peroxidase (DyP) in forest floor samples. Our results demonstrate that a wide range of peroxidases are produced by fungi inhabiting forest floor, and they are likely mediators of wood and litter decomposition and soil organic matter transformations.

However, there are obstacles to overcome. Primers used in this study mainly targeted basidiomycete sequences; inasmuch, our phylogenetic analyses of the newly gathered environmental sequences clustered them among known basidiomycete references. Nevertheless, sequences from the here analyzed environmental samples showed considerable diversity and were present in different clades of the calculated phylogenetic trees (Figs. 2, S1, S2, S3). Moreover, the finding of widespread occurrence of peroxidase transcripts among soil inhabiting basidiomycetous fungi by using these primer sets (Table S1), is remarkable and underlines the key role this fungal phylum in litter decomposition in temperate forest ecosystems. To confirm the presence of peroxidases, we measured Mn-independent peroxidase (MiP) activities with ABTS and Mn-dependent peroxidase (MnP) by following the oxidation of Mn^2+^ to Mn^3+^ directly in selected forest litter samples (Table 1, 4); both peroxidase activities were found in most of the tested samples, which is in accordance with the transcript-level expression. However, the measurement of peroxidase activity in soil extracts is not always specific enough to distinguish between the different types of fungal secretory peroxidases [8]. General peroxidase activity, for example, is often measured using L-DOPA (L-3,4-dihydroxyphenylalanine) as substrate ([31]; Table 1), whereupon it has to be corrected by phenol oxidase activity (laccase and tyrosinase also oxidize L-DOPA but in the absence of hydrogen peroxide; [32]). Manganese oxidizing peroxidases (i.e. MnP and VP) can directly be measured by following the formation of Mn^3+^ malonate complexes (i.e., at 270 nm; [26], [27]), but high UV background absorption may affect the assay. Alternatively, they can be indirectly detected by following the oxidation of a suitable substrate (ABTS, vanillylacetone, MBTH/DMAB) in the presence of Mn^2+^ but, as in case of L-DOPA, these peroxidase activities have to be corrected by phenol oxidase activity as well as MiP activities [13], [26], [33], [34]. It must be noted that only few studies have considered these different activities [35], [36]. Simple enzymatic tests for the specific detection of fungal MiP activities (i.e., of UPO and DyP, but theoretically also of LiP, Mn-independent activity of VP and hMnPs and phenol-oxidizing activity of generic peroxidases) in soils are not available so far and the existing specific assays can only detect relatively high UPO/DyP/LiP titers as typically observed in fungal pure cultures, e.g. during growth on saw dust [26]. All these critical points need to be taken into consideration, when measuring soil enzymatic activities of peroxidases in field surveys.

In our survey, we also analyzed the transcriptional expression of pure cultures of several litter-decomposing basidiomycetes under laboratory conditions and found a considerable diversity of peroxidase genes. Some of the analyzed species, e.g., *C. nebularis*, *C. tuberosa* and *E. glandulosa* (a wood degrader), exhibited transcripts of all three (super)families of secretory peroxidases (MnPs, DyPs, UPOs). A number of other litter-decomposing fungi were found to contain expressed MnP genes, which supports the hypothesis that Mn oxidizing peroxidases are key enzymes of litter and humus degradation in forest floor [34], [37]. Mn-agar plate tests complemented the finding of MnP transcripts by the formation of dark-brown MnO_2_ rings and spots originating from MnP-activity [24]. A current limitation, however, is the still scarce availability of peroxidase gene references in databases. For example, as recently evaluated (mid 2013), PeroxiBase and GenBank contained 311 entries of class II peroxidases from wood-decay fungi *vs.* only 11 references from litter-decomposing species *vs.* 16 sequences from ectomycorrhizal fungi. When investigating litter and soil samples, there is the great need to increase the number of reference sequences of fungi inhabiting these environments, and we assume that the majority of expressed peroxidases found in this study originate from basidiomycetous litter decomposers.

The identification of several peroxidase genes from *Mycena* spp. in forest floor samples supports this assumption. *Mycena* spp. have key functions in certain forest ecosystems, i.e. in litter decay, as they deploy the here analyzed peroxidases (MnPs, UPOs) and contain also several laccase genes, which were identified in previous surveys of litter samples from a beech forest in Germany (e.g., [38]). Using peroxidases and laccase to produce reactive radical intermediates in coordinated action, *Mycena* spp. may have a strong impact on the conversion of aromatics, and by extension, on humus turnover in forest soils [39].

Aside from saprotrophic species, there are also first indications for a contribution by ectomycorrhizal species to the peroxidase pool, because one environmental sequence matched to several class II peroxidase references of *Cortinarius* spp. This preliminary finding supports the hypothesis of Bödecker and coworkers [16] that ectomycorrhizal species may use MnP-like peroxidases for the mobilization of nutrients (nitrogen) in soils. Presently, we cannot exclude the involvement of these enzymes in carbon or nitrogen cycling, because their action might be dependent on the particular ecological niche (i.e. soil *vs.* litter). Moreover, it needs to be established that *Cortinarius* spp. in fact encode active MnP enzymes by characterization of respective proteins and activities. So far, we were not able to verify a MnP transcript-level expression or MnP activities in *C. odorifer* pure cultures (Kellner, *unpublished result*).

The class II peroxidases analyzed here represent the most investigated fungal peroxidases [3], [40]. Class II peroxidases cluster according to their biochemical features and sequences into “more or less” distinct groups, i.e. those of LiP, VP, MnP (long, short) and generic peroxidases (groups A–C) [4]. The majority of the environmental sequences found here contained an aspartic acid residue (Asp-175 in *P. chrysosporium* MnP1, Fig. 2) that is together with two other acidic residues (Glu35, Glu39; both not included in the amplified region) important for MnP activity (forming together with a heme propionate the Mn binding site). After phylogenetic analysis, most of these sequences appeared in appropriate groups, i.e., in group A outside of the true LiPs (group A.1) or in group B that contains the so-called long MnPs of the *P. chrysosporium* type [4]. A few environmental sequences do not have this Asp but display a putative catalytic tryptophan (Trp-171 in *P. chrysosporium* LIPH8, Fig. 2) that is characteristic for LiPs. However, the phylogenetic analysis has placed these sequences away from typical LiP sequences (group A.1). At the moment, we do not know the potential function of these “untypical” LiPs, but we assume that the class II peroxidase phylogeny will become more complex with novel upcoming sequences.

The deduction of potential peroxidase functions based on single key amino acid residues in partial sequences is a huge step forward towards functional interpretation of sequence surveys, especially when also respective enzymatic activities can be measured (e.g., MnP activity based on the formation of Mn^3+^-complexes or subsequent oxidation of indicator substrates). Unfortunately, linking of key amino acids is not yet possible for the different (sub)groups of heme-thiolate peroxidases (HTPs including UPOs) and DyPs [3], [41]; the identification of characteristic catalytic residues in our partial DyP sequences may be a first step to overcome this limitation. In this respect, UPOs are of particular interest, because enzymes of this type have unique properties, namely the catalysis of diverse oxyfunctionalizations including hydroxylations as well as *O*- and *N*-dealkylations [8]. Such reactions, which are usually catalyzed by intracellular monooxygenases (P450 enzymes; [42]), may be of general ecological and biotechnological interest, although their actual physiological function is not fully understood. Thus, a residue-based functional analysis of HTP/UPO sequences would be desirable, but is currently impossible because only a few types of these enzymes have so far been characterized (e.g. [43]). On the other hand, the widespread expression of this peroxidase type in forest litter samples indicates a strong role in ecologically relevant processes, perhaps in soil organic matter transformation as suggested for *A. bisporus* [44].

This study was designed to provide initial insight into the transcript-level activity on a broad, widespread basis in the community of fungi inhabiting forest floor. In this respect, we indeed found the expression of all three fungal secretory peroxidase (super)families across large forested areas as well as in abundant numbers. At several of these research sites, ecological treatments have been applied and again MnPs and UPOs were consistently found. Further, few identical peroxidase sequence types were found expressed in distantly located areas, which indicate that identical or highly similar taxa are present and perhaps responsible for decomposition processes. A similar result was found for partial fungal cellobiohydrolase genes *cbhI* amplified using DNA extracted from soil in several US research sites, nevertheless each site harboured a distinct fungal community [45]. Subsequent studies should include an in depth analysis of peroxidase gene richness and the transcription intensity by quantitative PCR, in concert with conclusive enzyme activity measurements to provide a clear understanding of ecosystem processes related to lignin degradation and humus formation. For example, in a previous survey dealing with sample sites overlapping with those studied here, Edwards and coworkers [15] found a strong down-regulation of fungal laccase genes under chronic atmospheric nitrogen deposition and there is some evidence that peroxidases may behave in a similar way [46], [47]. Thus, by combining different approaches with deeper phylogenetic analysis, it will be possible in future to more precisely define how these genes regulate ecosystem-level processes in forests.

## Supporting Information

Figure S1 **Phylogenetic analysis of class II peroxidases from basidiomycetes.** The phylogeny was inferred using the Neighbor-Joining method. The optimal tree with the sum of branch length = 41.32300055 is shown. The percentage of replicate trees in which the associated taxa clustered together in the bootstrap test (1,000 replicates) is shown next to the branches. The evolutionary distances were computed using the Poisson correction method and are in the units of the number of amino acid substitutions per site. The analysis involved 440 amino acid sequences. There were a total of 148 positions in the final dataset. Classification and annotation of larger groups shown in green followed Lundell and coworkers [4], in red: single genome references followed Floudas and coworkers [3], and GenBank entries in blue. In orange: peroxidases from litter and soil saprotrophs, green: ectomycorrhizal fungi, black bold: environmental sequences of own and previous studies. Transcriptional expressed own new reference sequences are underlined.
(JPG)

Figure S2 **Phylogenetic analysis of fungal unspecific peroxygenases (UPOs).** The evolutionary history was inferred using the Neighbor-Joining method. The optimal tree with the sum of branch length = 28.87498149 is shown. The percentage of replicate trees in which the associated taxa clustered together in the bootstrap test (1,000 replicates) is shown next to the branches. The evolutionary distances were computed using the Poisson correction method and are in the units of the number of amino acid substitutions per site. The analysis involved 151 amino acid sequences. There were a total of 98 positions in the final dataset. In orange: peroxidases from litter and soil saprotrophs, green: ectomycorrhizal fungi, black bold: environmental sequences. Transcriptional expressed own new reference sequences are underlined.
(JPG)

Figure S3 **Phylogenetic analysis of fungal dye-decolorizing peroxidases (DyPs).** The evolutionary history was inferred using the Neighbor-Joining method. The optimal tree with the sum of branch length = 33.91019168 is shown. The percentage of replicate trees in which the associated taxa clustered together in the bootstrap test (1,000 replicates) is shown next to the branches. The evolutionary distances were computed using the Poisson correction method and are in the units of the number of amino acid substitutions per site. The analysis involved 160 amino acid sequences. There were a total of 242 positions in the final dataset. In orange: peroxidases from litter and soil saprotrophs, green: ectomycorrhizal fungi, black bold: environmental sequences. Transcriptional expressed own new reference sequences are underlined.
(JPG)

Table S1 **Fungal cultures used in the study, and expressed peroxidase gene fragments and MnP activity found.**
(DOCX)
