# Supplementary figures and images for: Widespread Occurrence of Expressed Fungal Secretory Peroxidases in Forest Soils
Source: PLoS One. 2014 Apr 24;9(4):e95557. doi: 10.1371/journal.pone.0095557 (PMC3999038; doi:10.1371/journal.pone.0095557)

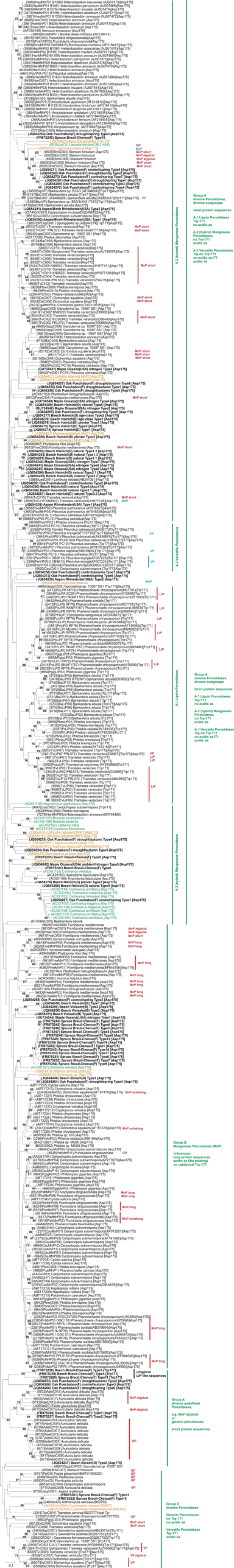

Supplement: Figure S1 — Phylogenetic analysis of class II peroxidases from basidiomycetes. The phylogeny was inferred using the Neighbor-Joining method. The optimal tree with the sum of branch length = 41.32300055 is shown. The percentage of replicate trees in which the associated taxa clustered together in the bootstrap test (1,000 replicates) is shown next to the branches. The evolutionary distances were computed using the Poisson correction method and are in the units of the number of amino acid substitutions per site. The analysis involved 440 amino acid sequences. There were a total of 148 positions in the final dataset. Classification and annotation of larger groups shown in green followed Lundell and coworkers [4], in red: single genome references followed Floudas and coworkers [3], and GenBank entries in blue. In orange: peroxidases from litter and soil saprotrophs, green: ectomycorrhizal fungi, black bold: environmental sequences of own and previous studies. Transcriptional expressed own new reference sequences are underlined. (JPG) [file pone.0095557.s001.jpg]

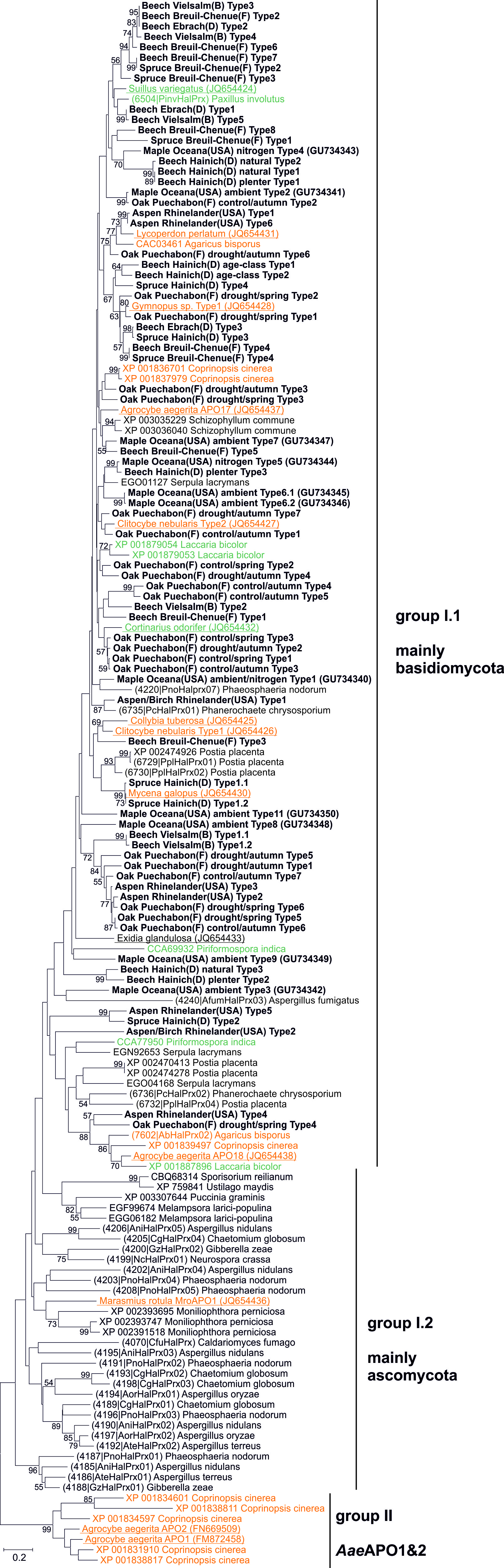

Supplement: Figure S2 — Phylogenetic analysis of fungal unspecific peroxygenases (UPOs). The evolutionary history was inferred using the Neighbor-Joining method. The optimal tree with the sum of branch length = 28.87498149 is shown. The percentage of replicate trees in which the associated taxa clustered together in the bootstrap test (1,000 replicates) is shown next to the branches. The evolutionary distances were computed using the Poisson correction method and are in the units of the number of amino acid substitutions per site. The analysis involved 151 amino acid sequences. There were a total of 98 positions in the final dataset. In orange: peroxidases from litter and soil saprotrophs, green: ectomycorrhizal fungi, black bold: environmental sequences. Transcriptional expressed own new reference sequences are underlined. (JPG) [file pone.0095557.s002.jpg]

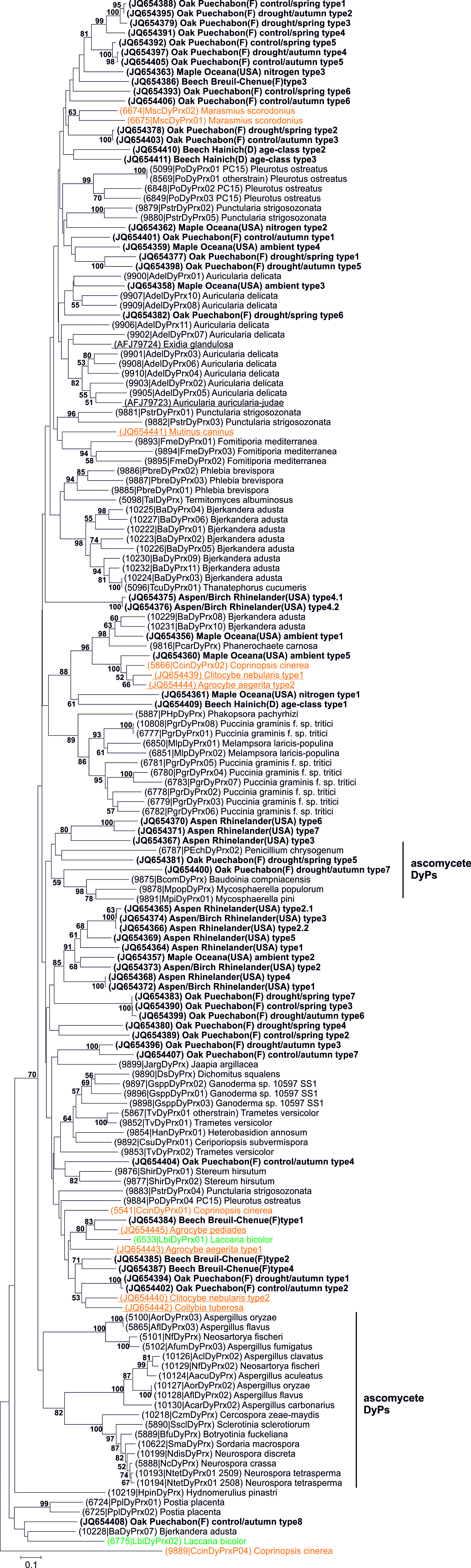

Supplement: Figure S3 — Phylogenetic analysis of fungal dye-decolorizing peroxidases (DyPs). The evolutionary history was inferred using the Neighbor-Joining method. The optimal tree with the sum of branch length = 33.91019168 is shown. The percentage of replicate trees in which the associated taxa clustered together in the bootstrap test (1,000 replicates) is shown next to the branches. The evolutionary distances were computed using the Poisson correction method and are in the units of the number of amino acid substitutions per site. The analysis involved 160 amino acid sequences. There were a total of 242 positions in the final dataset. In orange: peroxidases from litter and soil saprotrophs, green: ectomycorrhizal fungi, black bold: environmental sequences. Transcriptional expressed own new reference sequences are underlined. (JPG) [file pone.0095557.s003.jpg]
