# Supplementary material for: Widespread Occurrence of Expressed Fungal Secretory Peroxidases in Forest Soils
Source: PLoS One. 2014 Apr 24;9(4):e95557. doi: 10.1371/journal.pone.0095557 (PMC3999038; doi:10.1371/journal.pone.0095557)
Supplement: Table S1 — Fungal cultures used in the study, and expressed peroxidase gene fragments and MnP activity found. (DOCX) [file pone.0095557.s004.docx]

Supplementary Table 1. Fungal cultures used in the study, and expressed peroxidase gene fragments and MnP activity found.

| **Species, strain number** | **Origin** | **Expressed peroxidase genes** | **MnP plate test positive?** |
| --- | --- | --- | --- |
| *Agrocybe aegerita* DSM-22459 | Jena, Thuringia, Germany; col. Gramss | UPO, DyP | No |
| *Agrocybe pediades* CBS 101.39 | col. Quintanilha, Dec 1939 | MnP, DyP | Yes |
| *Clitocybe nebularis* IHI 460 | Hainich Mts., Thuringia, Germany; col. Kapturska; 10.10.2008 | MnP, UPO, DyP | No |
| *Collybia tuberosa* IHI 452 | Hainich Mts., Thuringia, Germany; col. Kapturska; 10.10.2008 | MnP, UPO, DyP | Yes |
| *Cortinarius odorifer* CBS 517.95 | Junkersbueterwald, Switzerland; col. Egli; 26.9.1985 | UPO | No |
| *Exidia glandulosa* DSM-1012 | Halle, Sachsen-Anhalt, Germany | MnP, UPO, DyP | No |
| *Gymnopus* sp. IHI 363 | Finland; col. Steffen | MnP, UPO | No |
| *Lycoperdon perlatum* IHI 456 | Hainich Mts., Thuringia, Germany; col. Kapturska; 10.10.2008 | UPO | Yes |
| *Macrolepiota procera* IHI 418 | isolated from commercial culture | MnP | No |
| *Marasmius rotula* DSM-25031 | Senftenberg, Brandenburg, Germany; col. Gröbe | UPO | No |
| *Mutinus caninus* IHI 508 | Ann Arbor, Michigan, USA; col. Kellner; 2008 | MnP, DyP | Yes |
| *Mycena epipterygia* IHI 195 | Finland; col. Steffen; Nov 2007 | MnP, DyP | No |
| *Mycena galopus* IHI 376 | Finland; col. Steffen | MnP, UPO | No |
| *Suillus variegatus* IHI 503 | - | UPO | No |
